# Natural Polymorphism in BUL2 Links Cellular Amino Acid Availability with Chronological Aging and Telomere Maintenance in Yeast

**DOI:** 10.1371/journal.pgen.1002250

**Published:** 2011-08-25

**Authors:** Elizabeth X. Kwan, Eric Foss, Leonid Kruglyak, Antonio Bedalov

**Affiliations:** 1 Clinical Division, Fred Hutchinson Cancer Research Center, Seattle, Washington, United States of America; 2 Molecular and Cellular Biology Program, University of Washington and Fred Hutchinson Cancer Research Center, Seattle, Washington, United States of America; 3 Lewis-Sigler Institute for Integrative Genomics and Department of Ecology and Evolutionary Biology, Princeton University, Princeton, New Jersey, United States of America; 4 Department of Medicine, University of Washington, Seattle, Washington, United States of America; University of Southern California, United States of America

## Abstract

Aging and longevity are considered to be highly complex genetic traits. In order to gain insight into aging as a polygenic trait, we employed an outbred *Saccharomyces cerevisiae* model, generated by crossing a vineyard strain RM11 and a laboratory strain S288c, to identify quantitative trait loci that control chronological lifespan. Among the major loci that regulate chronological lifespan in this cross, one genetic linkage was found to be congruent with a previously mapped locus that controls telomere length variation. We found that a single nucleotide polymorphism in BUL2, encoding a component of an ubiquitin ligase complex involved in trafficking of amino acid permeases, controls chronological lifespan and telomere length as well as amino acid uptake. Cellular amino acid availability changes conferred by the BUL2 polymorphism alter telomere length by modulating activity of a transcription factor Gln3. Among the GLN3 transcriptional targets relevant to this phenotype, we identified Wtm1, whose upregulation promotes nuclear retention of ribonucleotide reductase (RNR) components and inhibits the assembly of the RNR enzyme complex during S-phase. Inhibition of RNR is one of the mechanisms by which Gln3 modulates telomere length. Identification of a polymorphism in BUL2 in this outbred yeast population revealed a link among cellular amino acid availability, chronological lifespan, and telomere length control.

## Author Summary

Dietary restriction promotes longevity in many species, ranging from yeast to primates, and delays aging-related pathologies including cancer in rodent models. There is considerable interest in understanding how nutrient limitation mediates these beneficial effects. Much of what we have learned about the genetics of aging comes from studying isogenic model organisms, where the effects of single gene changes can be examined independently of other genetic alterations. In order to explore a broader spectrum of genetic variation and to gain insight into aging-related phenotypes as polygenic traits, we analyzed the chronological lifespan of 122 *S. cerevisiae* strains derived from a cross between laboratory and vineyard yeast strains. The major genetic locus controlling chronological lifespan was found to be identical to a previously mapped locus that controls telomere length. Identification of the responsible polymorphism in BUL2, a gene involved in controlling amino acid permeases, allowed us to establish a previously unrecognized link among cellular amino acid intake, chronological aging, and telomere maintenance. While human epidemiological studies have linked shortened telomeres with increased mortality, it is unclear how these processes are connected. Our results suggest that, in yeast, reduced amino acid uptake and consequent reduced nutrient signaling extend chronological lifespan but reduce telomere length.

## Introduction

The observation that dietary restriction promotes longevity in organisms ranging from yeast to primates raises the expectation that molecular mechanisms mediating this lifespan extension may also be shared among species. In support of the idea that related genetic circuitry controls aging in different species are the findings that genetic or pharmacological modulations of the conserved nutrient responsive pathways, such as target of rapamycin (TOR) [1] or insulin-like-growth factor (IGF-1) [2], increase lifespan in a wide range of species including mammals. The budding yeast *Saccharomyces cerevisiae* has become a popular model for studying the genetic and molecular basis for variation in lifespan. Two different forms of aging have been studied in yeast. Replicative lifespan (RLS) is defined by the number of daughter cells that are generated by a budding mother cell whereas chronological lifespan (CLS) is defined as the ability of yeast cells to survive in stationary phase as judged by the their capability to reenter the cell cycle after nutrients are reintroduced [3], [4]. The two types of aging in yeast are thought to have their counterparts in mammals as the aging of dividing stem cells or the aging of non-dividing cells such as neurons or muscle cells, respectively. In addition to replicative and chronological aging, mutant yeast cells dividing in the absence of telomerase components exhibit loss of viability [5] similarl to replicative senescence of human fibroblasts in culture [6].

Recent epidemiological studies of human populations demonstrated a correlation between reduced leukocyte telomere length and overall mortality [7], suggesting a link between telomere maintenance and organismal aging. Furthermore, life stress has been shown to influence leukocyte telomere length [8], establishing a role for environmental stress in telomere stability. Little is known about how these processes connect, though twin studies suggest that both telomere length regulation and longevity in humans have a strong genetic component [9], [10].

Most of what we have learned about telomere maintenance mechanisms and the genetics of aging comes from model organisms where the effects of the single gene changes can be examined independently from other genetic alterations. However, because natural populations are genetically diverse, differences in aging and telomere maintenance are more likely to result from the integration of effects of polymorphisms at multiple loci. In order to gain insight into telomere maintenance in genetically diverse populations, we have previously employed an outbred yeast model consisting of 122 haploid progeny derived by a cross of vineyard RM11-1a (RM) and laboratory S288c yeast (BY) [11]. Parental strains differ at 0.5% of their nucleotides and the progeny have been genotyped at >3000 markers, allowing for quantitative trait locus (QTL) mapping. In a previous telomere length study, we identified several loci that control telomere variation in this cross [12].

In this study, we used the same outbred model to explore chronological aging as a complex trait. During the course of these studies, we found that that one of the loci that controls chronological lifespan is identical to a major locus found to control telomere length, suggesting a previously unrecognized link between the two yeast aging-related phenotypes. This was an intriguing finding because changes in telomere length are linked to DNA replication, while chronological aging occurs in non-dividing cells. Furthermore, the two phenotypes were regulated in opposite directions by this locus: strains that inherited the vineyard allele had shorter telomeres and longer lifespans. We found that a single amino acid substitution in Bul2, a component of an ubiquitin ligase complex which polyubiquitylates amino acid permeases and regulates their presence at the cell membrane, controls cellular amino acid availability and is responsible for the variation in both telomere length and CLS. We also elucidated a pathway by which decreased cellular amino acid uptake conferred by the BUL2 polymorphism and the consequent inhibition of nutrient-responsive TOR1 signaling lead to reduced telomere length.

## Results

### Regulation of chronological lifespan is a dynamic process controlled by many loci

**Figure 1:**
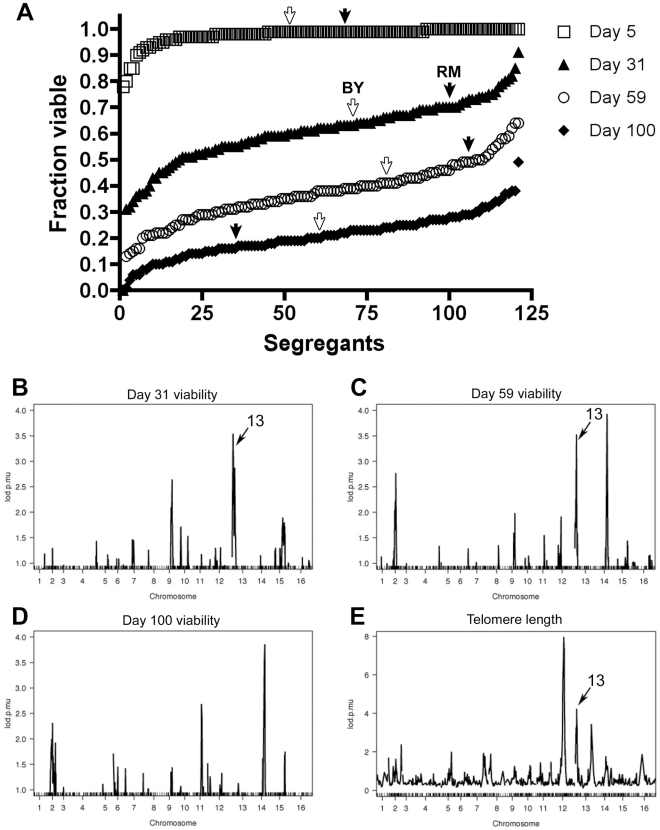
Genomic linkage of chronological lifespan in *S. cerevisiae* segregants. (A) Stationary phase viabilities of the segregants and parental strains (denoted by arrow) at different timepoints. Genome-wide linkage scans for viability after (B) 30 days, (C) 59 days, (D) 100 days and for (E) telomere length. Linkage to locus in common for telomere length and chronological lifespan on chromosome 13 is denoted by arrow.

To determine chronological lifespan of the 122 haploid progeny (segregants) from the RM/BY cross, strains were grown in YPD medium in 96-well plates to stationary phase, where cells maintain metabolic activity but cease mitotic division. Chronological lifespan (CLS) studies are often done in synthetic media, where yeast lifespans can be analyzed in a few weeks [13]. Because of the observation that the use of synthetic medium in CLS studies exposes cells to lifespan-limiting acidification [14], we decided to carry out segregant CLS analysis in YPD where acidification of the media during culture outgrowth is not a problem. After intervals of approximately 30 days, we harvested 1 µL of each stationary phase culture, spotted culture dilutions on YPD plates, and determined viability of cultures as the ratio of microcolonies after 24 hours of growth to the total cell number plated. We found excellent correlation (R = 0.98) between the cell viability determined using our microcolony method and the viability measured using colony forming ability (Figure S1). The vast majority of cultures were found to be fully viable after the initial interval of 5 days in stationary phase (Figure 1A). Further along in stationary phase, segregant culture viabilities decreased to an average of 70% after 31 days (range 30–91%), 35% after 59 days (range 11–62%) and 20% after 100 days (range 0.5–40%). The observed viability distributions of the chronologically aged segregant strains displayed several interesting features. First, the variation in viability between segregants was continuous, suggesting that multiple genetic loci control survival among the segregants. Second, we observed that the parental strains' phenotypes are in the middle of the range. Such transgressive segregation, in which the segregant progeny exhibit more extreme phenotypes than either parental strain, suggests the presence of compensatory genetic loci within both the RM and BY parental backgrounds. Finally, the rank of the segregant viabilities was not static, as illustrated by the changing order of the parental strains over time, which suggests that different genes are responsible for early and late viabilities.

We used genome-wide linkage analysis to identify the loci (QTL) responsible for the variation in chronological lifespan. Each segregant strain has been characterized for BY or RM inheritance at 2,956 polymorphic markers across the genome [11]. Using genome-wide linkage analysis, phenotype distributions can be compared between segregants that inherit the BY or RM sequence at each locus. A significant difference between the two distributions establishes a linkage between the trait of interest and the genomic sequence near the tested polymorphic marker. We found that stationary phase survival is linked to several genetic loci, consistent with the observed continuous range in viability (Figure 1B–1D, Table S2). We also noticed that the strength of linkage of the mapped loci changes with time. The chromosome 13 linkage, for instance, has LOD scores >3.5 at 31 and 59 days, yet it has no role in controlling viability after 100 days in culture. On the other hand, the chromosome 14 linkage had the opposite temporal pattern: not significant at day 31 yet has LOD scores >3.5 at day 59 and 100. The alteration of the relative importance of different loci at different time points suggests that cells depend on different cellular processes during early and late stages of chronological lifespan.

### Chronological lifespan, telomere length, and cellular permease activity are linked to the same polymorphism in BUL2

Comparison between the genome scan for loci that control chronological lifespan and our previous analysis for loci that control telomere length (Figure 1E) revealed that the strongest linkage for chronological lifespan at day 31 (chromosome 13 locus) is congruent with a previously identified locus that controls telomere length [12]. The segregant strains which inherited the RM allele of chromosome 13 locus had longer CLS (65% versus 56% viability at 30 days) and shorter telomeres (261 bp versus 286 bp) compared to strains which inherited the BY allele of the locus. In order to determine whether other mutants with short or long telomeres exhibit either reciprocal effects or alterations in CLS in general, we examined a panel of deletion mutants known to have telomere length alterations and found no correlation between telomere length changes and CLS (Figure S2). Likewise, a more general comparison of CLS and telomere length, using data from the recent global CLS study [15] and our previous telomere length screen [12], did not reveal any correlation between telomere length and CLS (Figure S2). While we found no general correlation between telomere length and CLS, the striking overlap of genetic linkage between telomere length and chronological aging in this cross led us to hypothesize that these two traits are both controlled by a common polymorphism and that identifying the responsible gene may reveal an unexpected link between telomere maintenance and chronological aging.

**Figure 2:**
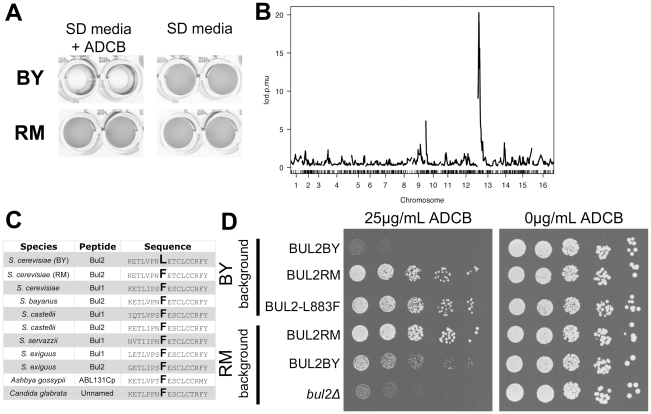
Chromosome 13 locus contains a loss of function polymorphism in BUL2. (A) The RM and BY parental strains show differential growth in media containing the toxic proline analog ADCB. (B) QTL mapping reveals that segregant sensitivity to ADCB is strongly linked to the same locus on chromosome 13 that controls chronological lifespan. (C) Alignment of amino acid sequences from Bul2 homologs identifies a leucine substitution of a conserved phenylalanine residue at position 883 conferred by the BY allele of BUL2 (T2647C). (D) Full BUL2 RM allele replacement and single nucleotide point mutation encoding the BUL2 L883F substitution both confer ADCB resistance in the BY strain background. BUL2BY allele replacement or deletion of BUL2 decreases growth of RM strains in media containing ADCB.

Among the polymorphisms in the mapped region, we identified one in the coding region of BUL2, a gene encoding a component of the Rsp5p E3-ubiquitin ligase complex involved in amino acid permease sorting. During growth in the presence of rich nitrogen sources, high affinity amino acid permeases, such as the general amino acid permease GAP1 and the proline transporter PUT4, are polyubiquitylated by a complex consisting of Bul1, Bul2 and Rps5, which specifies vacuolar-targeting of permeases for degradation [16], [17]. Cellular amino acid permease activity can be monitored using the toxic proline analogue ADCB, which is transported across the cell membrane via nitrogen-regulated PUT4 and GAP1 [18]. We found that the parental RM and BY strains exhibit a striking difference in ADCB sensitivity when grown with a rich nitrogen source (Figure 2A). Consistent with higher permease activity and amino acid intake relative to the RM strain, the BY strain was not able to grow at concentrations of ADCB that were non-toxic to the RM strain. Genome-wide linkage analysis of ADCB sensitivity in the segregants demonstrates that the BUL2-containing locus underlies the parental differences in permease activity (Figure 2B). The BY strain carries a single Leu883Phe substitution relative to the RM version of Bul2, which is conserved among many fungal homologs (Figure 2C) and all but three of the sequenced *S. cerevisiae* strains (F883L is present in S288c and the two baking isolates YS2 and YS9) [19]. Engineering the RM allele of BUL2 into the BY strain restored ADCB resistance, whereas substitution of the BY BUL2 allele into the RM strain resulted in ADCB sensitivity (Figure 2D). These findings indicate that the BY BUL2 Phe883Leu polymorphism confers a loss of Bul2 function, similar to that of a *bul2Δ* mutant, and increases permease activity and amino acid uptake.

**Figure 3:**
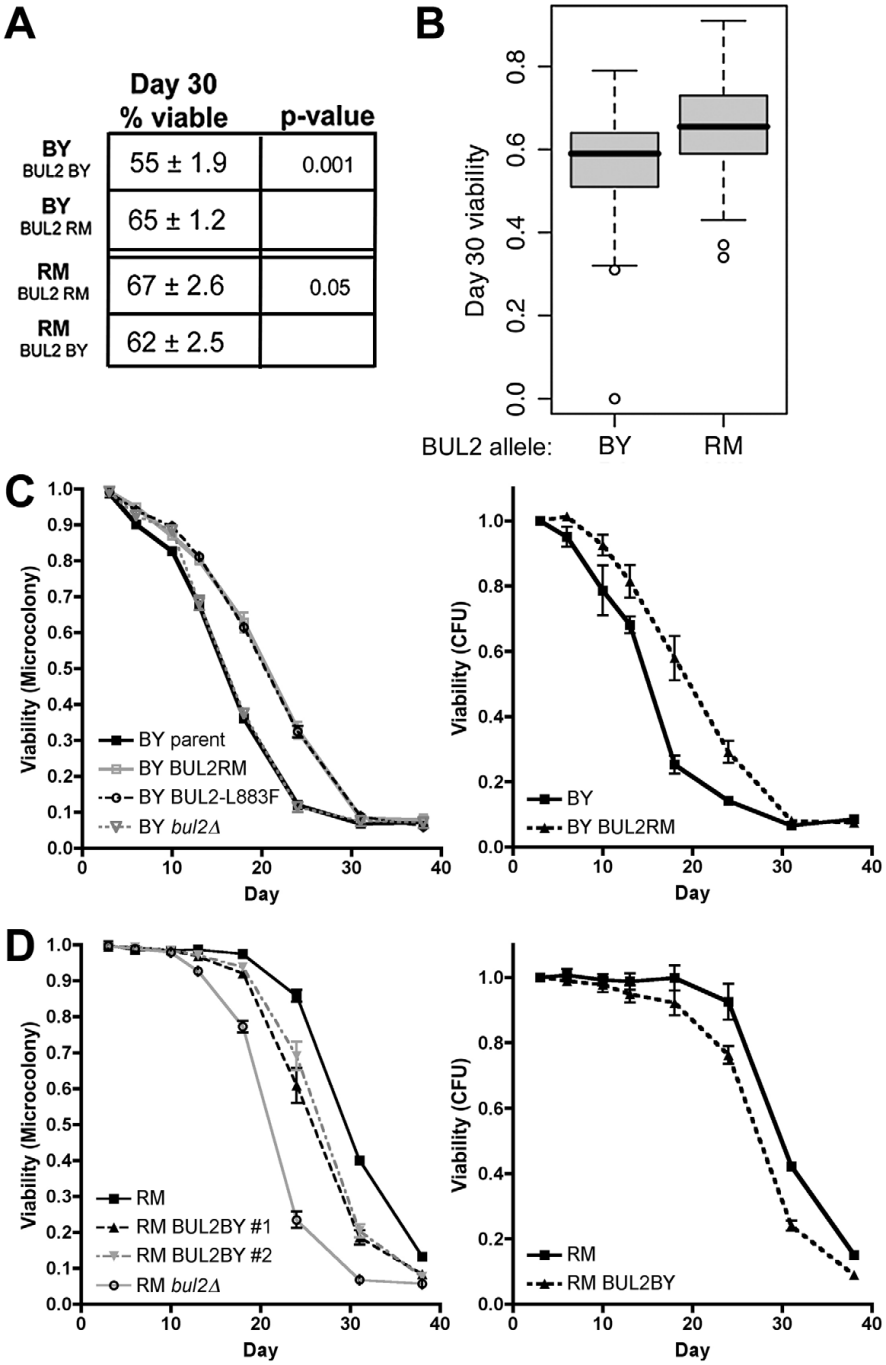
The BUL2 polymorphism regulates chronological lifespan. (A) Viability of the parental and BUL2 allele replacement strains after 30 days in stationary phase in YPD. (B) Viabilities of segregants after 30 days in stationary phase, separated on basis of BUL2 inheritance. Mean viability for segregant populations with BUL2BY and BUL2RM are 56% and 65% respectively (p = 6.1×10^−5^). (C) The BUL2RM allele replacement significantly extended longevity of the BY strain, measured by both microcolony counting (p≤0.05 at days 10, 13, 18 and 24) and colony formation (p≤0.05 at days 18 and 24). The BY *bul2Δ* strain exhibited CLS identical to the BY parental strain. (D) The RM parental strain had greater CLS than either the BUL2BY allele replacement (microcolony: p≤0.05 at days 18, 24, 31 and 38; CFU: p≤0.05 at days 24, 31 and 38) or BUL2 deletion strain (p≤0.05 at days 13, 18, 24, 31 and 38). For longevity curves in C and D, triplicate cultures were aged in buffered SC media.

We next evaluated whether the same BUL2 polymorphism that controls cellular permease activity also mediates chronological lifespan and telomere length variation. The replacement of BUL2 in the BY parental strain with the RM allele led to an increase in chronological lifespan (from 55% to 65% viable cells at 30 days in YPD medium), which was similar in magnitude to the increase in chronological lifespan conferred by the RM BUL2 allele in the segregants (Figure 3A, 3B). Conversely, the replacement of the RM BUL2 allele with the BY BUL2 allele in the RM parental strain decreased chronological lifespan (67% versus 62%) after 30 days. We next examined the effect of BUL2 alleles on the time-dependant viability curves in both laboratory and vineyard background using the synthetic media that is commonly used for CLS studies. (In order to minimize the viability reduction due to media acidification, we used buffered SC medium [14]). Consistent with previous reports, we observed that CLS is shortened in SC medium compared to YPD, however, restoration of BUL2 function using RM BUL2 allele in the laboratory strain extended chronological life span even more robustly than we have observed in YPD (Figure 3C). BUL2 replacement in the vineyard strain with the hypomorphic BUL2BY allele shortened CLS and deletion of BUL2 led to further reduction in CLS (Figure 3D), which parallels the effect of BUL2 allele replacement and BUL2 deletion on cellular permease activity in the vineyard strain, judged by increased ADCB sensitivity in the BUL2BY alleles and BUL2 deletion (Figure 2D). The effects of BUL2 allele replacement on CLS results were also confirmed using standard colony formation metrics [13]. These findings demonstrate that the BUL2 polymorphism controls variation of chronological lifespan in the RM/BY cross.

**Figure 4:**
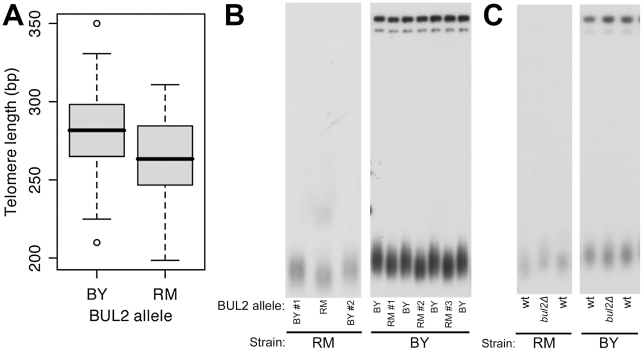
The BUL2 polymorphism is responsible for segregant telomere length phenotypes. (A) Segregant telomere lengths, separated by BUL2 inheritance. Mean telomere lengths of segregants with BUL2BY and BUL2RM are 286 basepairs and 261 basepairs respectively (p = 3.0×10^−4^). (B) Southern blot analysis comparing telomere length in multiple independent BUL2 allele replacement transformants and wildtype parental strains. Strains with the BY allele of BUL2 have longer telomeres in both parental backgrounds. (C) In the RM background, deletion of BUL2 increases telomere length. BUL2 deletion in the BY background has no effect on telomere length.

The average telomere length in the segregants that contain the BY allele of BUL2 was 286 bp, which is 25 bp longer than the telomere length average of segregants that contain the RM allele (261 bp) (Figure 4A). Therefore, if BUL2 is the responsible polymorphism for telomere length alteration, then the BUL2 allele replacement in the RM parental strain is expected to create a 25 bp increase in telomere length, while the allele replacement in the BY strain would have a modest telomere length reduction. We found that allele replacement of BUL2 in both parental strains led to alterations in telomere length as predicted by the segregant analysis: telomeres were found to be longer in the RM strains with BUL2 replaced by the BY allele and telomeres were shorter in the BY strains containing the RM BUL2 allele replacement (Figure 4B). As expected from the segregant analysis, the effect of allele replacement was modest, but also consistent and reproducible, as shown by analysis of several independent strains. Deletion of BUL2 lengthened telomeres in the RM background, but had no effect in the BY background (Figure 4C). These results demonstrate that the leucine residue substitution present in the BY parent creates loss of Bul2 function, leading to higher activity of amino acid permeases on cell membranes, reduced chronological lifespan, and increased telomere length.

### Telomere length alteration by Bul2 polymorphisms is mediated by Gln3

**Figure 5:**
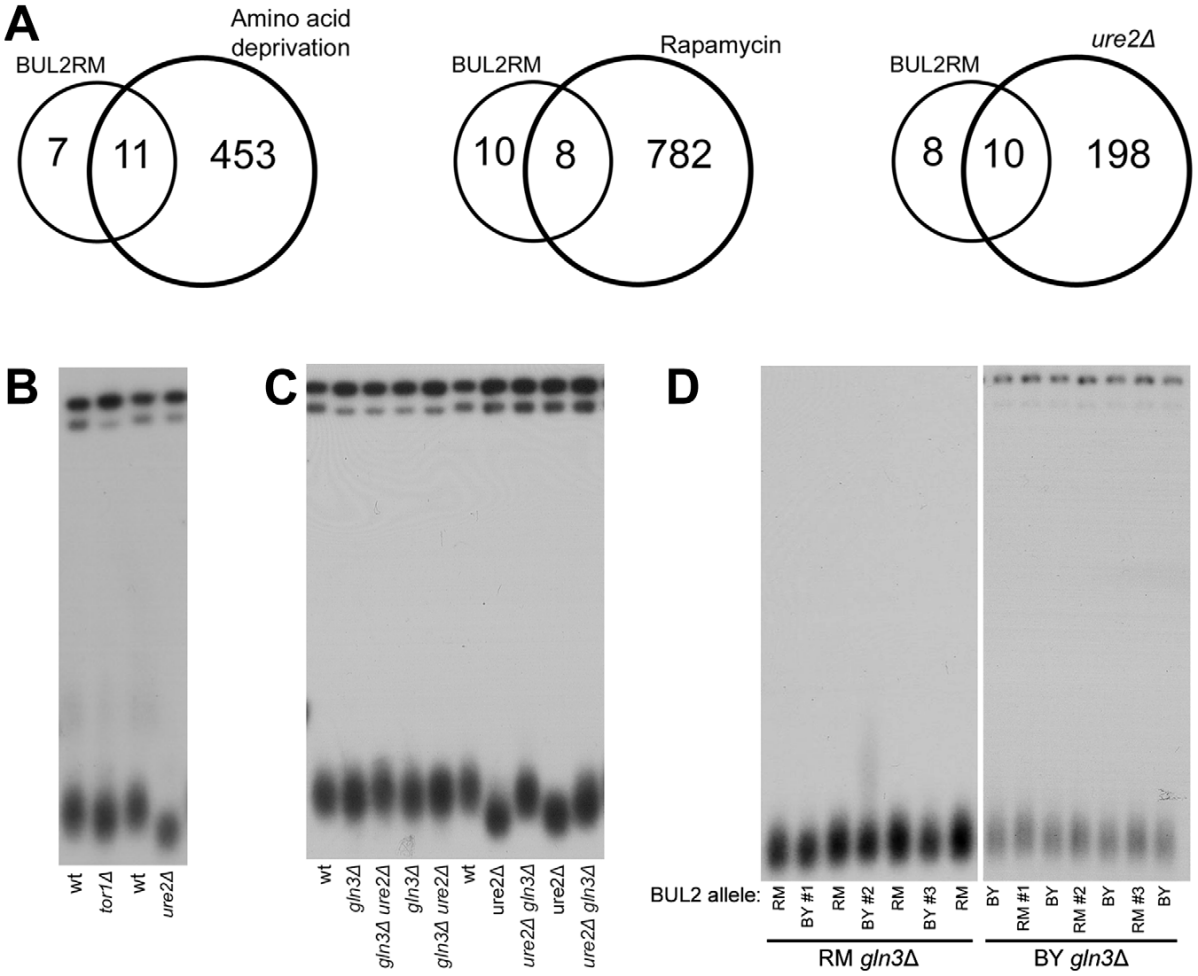
Transcriptional activator Gln3 mediates BUL2 allele-induced telomere length alterations. (A) Overlap between transcripts upregulated by the RM allele of BUL2 among the segregants and those which are upregulated by amino acid deprivation, rapamycin treatment, or the deletion of URE2 in the BY background. 11 of the 464 genes upregulated by amino acid deprivation (1 hour), 8 of the 796 genes upregulated by rapamycin treatment (1 hour), and 10 of the 208 genes that are upregulated in *ure2Δ* cells are among the18 genes that are upregulated by the RM BUL2 allele (p = 1.1×10^−8^, p = 1.2×10^−3^, and p = 8.5×10^−11^ respectively for the probability of overlap occurring by chance). (18 of the 19 BUL2RM upregulated transcripts are represented in the amino acid deprivation, rapamycin, and *ure2Δ* transcript data). (B) *tor1Δ* and *ure2Δ* mutants in the BY background have shorter telomeres than wildtype cells. (C) Deletion of URE2 in the BY background shortens telomere length through a GLN3-dependent mechanism. *ure2Δ* mutant telomere length shortening is rescued by the deletion of GLN3. (D) Southern blot analysis comparing telomere length of multiple independent BUL2 allele replacement transformants and parental strains lacking GLN3. BUL2 allele replacement does not alter telomere length in parental strains lacking GLN3.

Reduced availability of cellular nitrogen and amino acids conferred by the restoration of Bul2 function is expected to reduce the activity of the nutrient sensitive TOR1 kinase. Since the region containing the BUL2 locus had been previously identified as a regulatory hotspot that controls abundance of many transcripts in this cross [20], we evaluated whether these transcriptional alterations could be mediated by alterations in TOR1 activity. Consistent with this possibility, we found that the set of genes overexpressed in strains containing BUL2RM significantly overlaps with genes that were found to be overexpressed in response to amino acid deprivation (p = 1.1×10^−8^) and rapamycin (p = 1.2×10^−3^) (Figure 5A, Table S3) [21], known inhibitors of TOR1 activity [22], [23]. Because reduction of TOR1 signaling has been shown to extend chronological lifespan [24], [25], the viability gain in chronological aging assays conferred by the restoration of Bul2 function can be explained by reduced activity of the nutrient responsive TOR pathway. Could the same gene network be mediating telomere length alterations conferred by BUL2 function?

To investigate this possibility, we re-examined data from our previous genome-wide telomere length screen [12], focusing on deletion mutants of genes in the nitrogen signaling circuit. We reasoned that such mutants would likely affect telomere length through the same mechanism as BUL2, thus we might gain insight into BUL2's mechanism of action on telomere length from known modes of action through these other nitrogen-signaling mutants. Among the mutants in genes involved in nitrogen signaling, we found that cells lacking TOR1 have modest reduction in telomere length and that cells lacking URE2 have strikingly short telomeres (Figure 5B). In rich nitrogen environments, Ure2 binds to the transcriptional activator Gln3 and inactivates it through cytoplasmic sequestration [26], [27]. Upon encountering nitrogen-limiting environments, Gln3 is released from its complex with Ure2 and translocates to the nucleus to upregulate nitrogen catabolite responses [28]. The short telomere phenotype in *ure2Δ* mutants is mediated by Gln3, as we found that the deletion of GLN3 restored the short telomere lengths in *ure2Δ* cells back to wildtype lengths (Figure 5C).

We hypothesized that the reduced nitrogen availability occurring in cells with functional Bul2 (i.e. the RM allele) leads to increased Gln3 transcriptional activity and shorter telomeres. In order to evaluate whether transcriptional alterations previously mapped to the region containing the BUL2 locus [20] could be mediated by Gln3, we compared the set of genes that are upregulated by the RM BUL2 allele with the genes that are upregulated in response to URE2 deletion. Of the 19 transcripts that are significantly upregulated in strains with the RM BUL2 allele, 10 transcripts were found to be overexpressed in our transcript array analysis of *ure2Δ* cells (of which there were 208 transcripts), including known direct Gln3 targets such as BAT2 and DIP5 (Figure 5A, Table S4) (p = 8.5×10^−11^) [29]. These findings, along with previous reports which link loss of Bul2 to decreased Gln3 nuclear localization [30], support a model in which restoration of Bul2 function leads to decreased cellular nitrogen availability, thereby promoting Gln3 transcriptional activity and reduction of telomere length.

Could Bul2's effect on telomere length be mediated by Gln3? To address this question, we examined the effect of the BUL2 allele replacement in cells lacking GLN3. We found that neither did the RM BUL2 allele in the BY *gln3Δ* strain shorten telomeres, nor did the BY allele replacement increase telomere length in the RM *gln3Δ* strain (Figure 5D). The requirement of Gln3 for BUL2 allele-induced telomere alterations supports the idea that BUL2 telomere length changes are mediated by modulation of Gln3 transcriptional activity. These findings, along with previous reports which link loss of Bul2 to decreased Gln3 nuclear localization [30], support a model in which restoration of Bul2 function leads to decreased cellular nitrogen availability, thereby promoting Gln3 transcriptional activity and reduction of telomere length.

### Gln3 modulates nuclear-cytoplasmic shuffling of ribonucleotide reductase components

In order to determine the relationship of the telomere maintenance defect caused by the deletion of URE2 to other pathways that participate in telomere maintenance, we compared telomere lengths of *ure2Δ* single mutants and double mutants that were *ure2Δ* and deficient in either DNA damage signaling (*tel1Δ*), telomerase (*tlc1Δ*), or telomere-capping (y*ku70Δ*) functions. The *ure2Δ* cells showed synthetic telomere length phenotypes with the y*ku70Δ*, *tel1Δ*, and *tlc1Δ* mutants (Figure S3), suggesting that Ure2's effect on telomere maintenance acts independently from pathways involved in telomere extension, telomere-capping, and TEL1-mediated DNA damage signaling.

**Figure 6:**
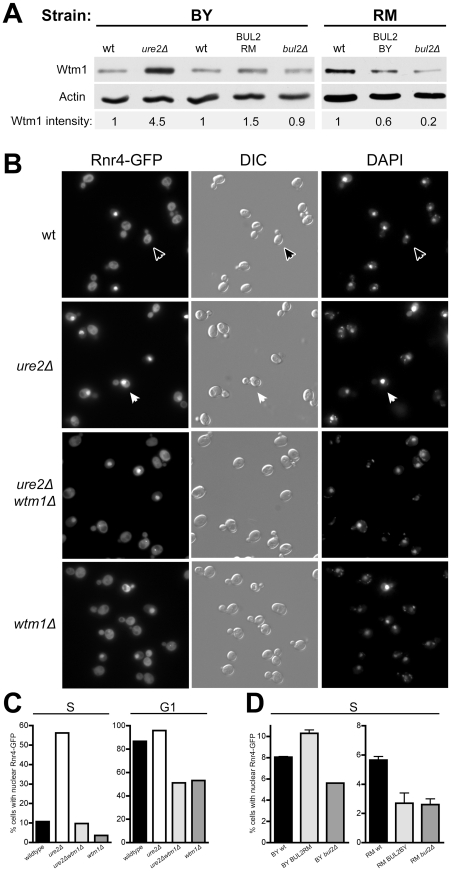
*ure2Δ* mutants and cells with BUL2RM have increased Wtm1 expression and activity. (A) Immunoblotting confirms that *ureΔ* mutants and strains with BUL2RM have increased expression of Wtm1, an inhibitor of ribonucleotide reductase. Wtm1 expression was normalized to actin and Wtm1 intensity is listed as relative to wildtype expression. (B,C) Wildtype budded (S-phase) cells have largely cytoplasmic Rnr4-GFP localization (black arrowhead), with nuclear exclusion of Rnr4-GFP, while *ure2Δ* mutants exhibit high fractions of budded S-phase cells with nuclear localization of Rnr4-GFP (white arrowhead). Increased nuclear retention of Rnr4-GFP in *ure2Δ* mutant is rescued by deletion of WTM1. Unbudded (G1) wildtype and *ure2Δ* cells both have primarily nuclear Rnr4-GFP localization, which is reduced by the deletion of WTM1. (D) Restoration of Bul2 function with the BUL2RM allele in the BY strain increases the number of cells with nuclear S-phase Rnr4-GFP (p = 0.02), while replacement with the hypomorphic BUL2BY allele in the RM strain results in fewer cells with S-phase nuclear Rnr4-GFP (p = 0.05). Deletion of BUL2 reduced the amount of cells with nuclear S-phase Rnr4-GFP in both the BY strain (p = 0.0002) and RM strain (p = 0.03).

Our previous study of telomere maintenance genes identified a significant subset of mutants involved in nucleotide biosynthesis as having altered telomere length [12]. For instance, loss of the ribonucleotide reductase large subunit RNR1 results in telomere shortening on par with loss of YKU70 or TEL1. Since nitrogen availability dictates growth, we speculated that mimicry of nitrogen starvation created by increased nuclear Gln3 would induce cells to conserve nitrogen and restrict nucleotide synthesis, and this in turn would cause shortening of telomeres. We first examined transcript levels in *ure2Δ* cells, anticipating reductions in nucleotide biosynthesis gene expression, but found only modest decreases in RNR1 and other nucleotide genes unlikely to account for the magnitude of telomere shortening in *ure2Δ* mutants. However, among the upregulated genes in *ure2Δ* cells, we found a strong increase in expression of Wtm1, an inhibitor of ribonucleotide reductase. Wtm1 protein levels were found to be almost 5-fold higher in *ure2Δ* cells compared to wildtype (Figure 6A). In addition, allele replacement with BUL2RM in the BY background gave rise to a 50% increase in Wtm1, while in the vineyards strain the replacement of BUL2 with the hypomorphic BUL2BY and BUL2 deletion decreased the Wtm1 protein level by 40% and 80% respectively (Figure 6A).

The ribonucleotide reductase complex assembles during S-phase and consists of large Rnr1 subunits and the two small subunits Rnr2 and Rnr4. Unlike Rnr1, which is always cytoplasmic, Rnr2 and Rnr4 are localized in the nucleus during G1 and translocate to the cytoplasm during S-phase [31]. This process is controlled by Dif1, which promotes nuclear import, and Wtm1, which anchors the small subunits Rnr2 and Rnr4 in the nucleus [32], [33]. Based on our observation that Wtm1 expression increases in *ure2Δ* cells, we hypothesized that *ure2Δ* cells have increased nuclear retention of the small subunits Rnr2 and Rnr4. As previously observed, we found that Rnr4-GFP is nuclear during G1 and cytoplasmic during S-phase in wildtype cells (Figure 6B, 6C). While Rnr4-GFP is appropriately nuclear in *ure2Δ* cells during G1, 56% of *ure2Δ* cells retain Rnr4-GFP in the nucleus during S-phase. We determined that this aberrant nuclear Rnr4 localization in *ure2Δ* is dependent on Wtm1 since *ure2Δwtm1Δ* double mutants have completely restored cytoplasmic localization of Rnr4-GFP. Rescue by WTM1 deletion is not merely due to loss of nuclear Rnr4 localization: more than 50% of *wtm1Δ* cells still maintain nuclear localization of Rnr4-GFP in G1 (Figure 6C). Examination of strains with different BUL2 alleles revealed that alteration of Bul2 function has a small but reproducible effect on S-phase Rnr4-GFP localization (Figure 6D). Both RM BUL2BY and RM *bul2Δ* strains had 2.6% of S-phase cells with nuclear Rnr4-GFP, which is a significant decrease from the 5.6% seen in the RM wildtype strain. The fraction of cells with nuclear Rnr4-GFP increases from 8.0% in BY wildtype to 10.3% in the BY strain with the RM allele of BUL2 and decreases to 5.6% of S-phase cells in the BY *bul2Δ* strain. These results suggest that cells with decreased TOR signaling, such as in *ure2Δ* mutants and cells with BUL2RM, form fewer ribonucleotide reductase complexes during S-phase due to increased Wtm1 expression.

**Figure 7:**
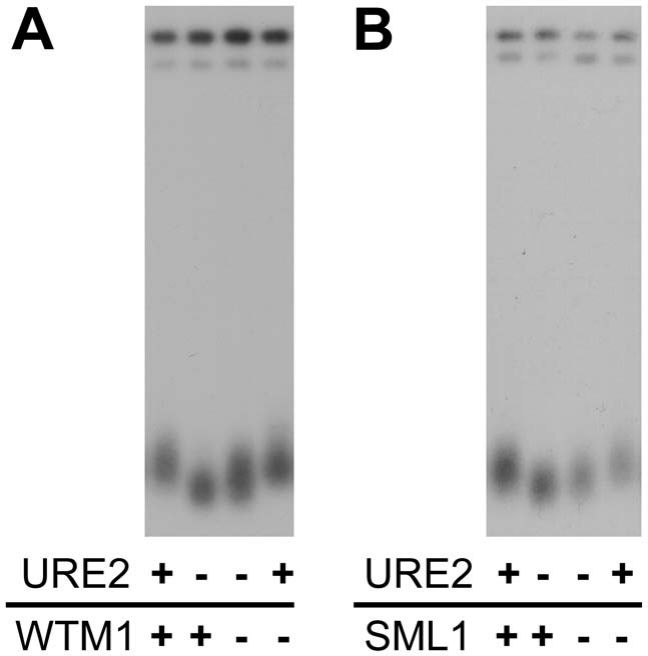
Removal of ribonucleotide reductase inhibition alleviates the short telomere phenotype in strains lacking URE2. Deletion of genes encoding ribonucleotide reductase inhibitors (A) WTM1 and (B) SML1 partially rescues telomere shortening conferred by deletion of URE2.

We then investigated whether deletion of WTM1 would rescue the *ure2Δ* telomere length shortening. Telomere length comparison of *ure2Δ* and *ure2Δwtm1Δ* mutants reveals that deletion of WTM1 partially rescues telomere shortening due to loss of URE2 (Figure 7A). Along the same lines, we found that deletion of the Rnr1 inhibitor SML1 [34] also abrogates the *ure2Δ* short telomere length defect (Figure 7B). These findings support our hypothesis that the shortened telomeres in *ure2Δ* cells are due, at least in part, to limitation of ribonucleotide reductase activity.

## Discussion

**Figure 8:**
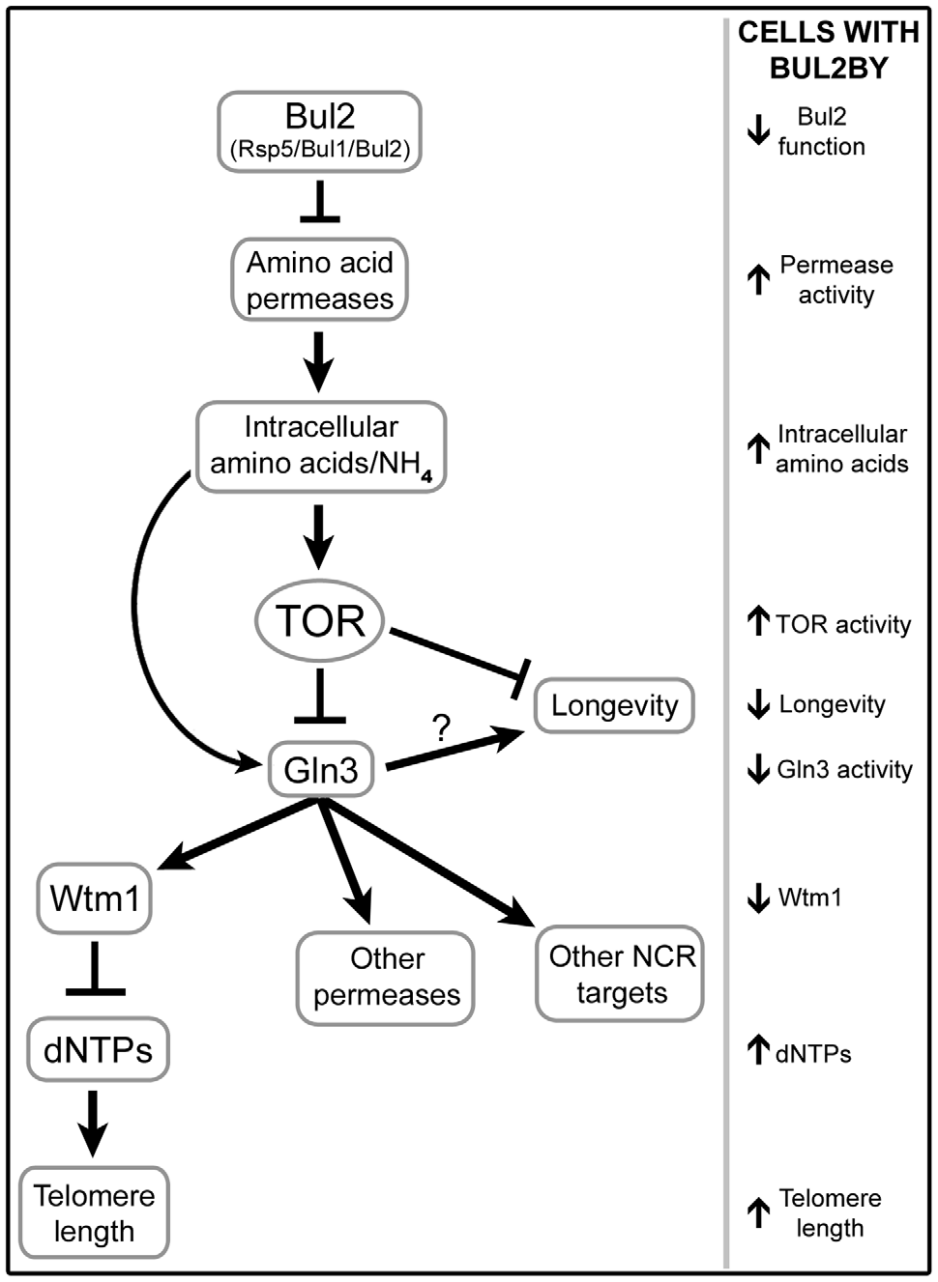
Model for BUL2-mediated alteration of telomere length and chronological lifespan.

Examination of quantitative trait loci that regulate chronological aging and telomere length in the progeny from a cross between the laboratory strain S288c and a vineyard strain, RM11-1a, led to identification of a polymorphism in BUL2 which alters trafficking of amino acid permeases and cellular amino acid import. Loss of Bul2 function, conferred by the laboratory allele of the gene, initiates a cascade of events outlined in Figure 8 that centers on TOR, a nutrient-responsive protein kinase previously implicated in CLS control. Our study defines a novel downstream role for TOR signaling in the regulation DNA replication and telomere maintenance through Gln3-mediated assembly of ribonucleotide reductase during S-phase.

Amino acids are powerful activators of TOR signaling not only in yeast, but also in multicellular eukaryotes. For *Drosophila melanogaster* larvae, amino acid deprivation inhibits TOR activity and leads to growth inhibition and reduced body size [35]. Similarly, *Caenorhabditis elegans* lacking the intestinal amino acid transporter pep-2 also have reduced body size [36]. Increasing evidence suggests that reduced intake of amino acids, which consequently reduces TOR activity, may be a key component of life-extending dietary interventions. Lifespan extension granted by dietary restriction in *D. melanogaster* was abolished by re-addition of amino acids [37]. Additionally, dietary reduction of a single essential amino acid, either tryptophan or methionine, was sufficient to confer lifespan extension in both mice and rats [38]–[40]. While dietary restriction studies in *S. cerevisiae* typically involve glucose restriction, our finding that restoration of Bul2 function and resulting reduction of cellular amino acid import extends CLS supports the idea that amino acid-mediated regulation of TOR signaling controls longevity.

While several of the upstream molecular events that control TOR activity, such as growth factors and energy status, are understood in great detail [41], we only have rudimentary knowledge of how cells sense amino acid sufficiency and transmit this signal to TOR. TOR forms two separate complexes: the rapamycin-sensitive TOR complex 1 (TORC1), which regulates growth, ribosome biogenesis, translation and lifespan, and the rapamycin-insensitive TOR complex 2 (TORC2) involved in actin cytoskeleton organization and cell wall integrity [42]. Recent studies in mammalian cells have identified several components that are required for TOR activation by amino acids, including Rag GTPase heterodimers involved in the recruitment of TORC1 complex to the lysosomal membrane compartment [43]. In addition to their roles as activators of TOR, the *S. cerevisiae* Rag GTPase orthologs Gtr1 and Gtr2 [23] are also implicated in the retrieval of Gap1 and other high affinity amino acid permeases from the vacuolar trafficking pathway [44], thus promoting their localization to the plasma membrane. Because the retrieval of Gap1 from the vacuolar targeting pathway is regulated by amino acid availability (discussed below), these findings raise the possibility that the related amino acid-responsive pathway that controls TOR also controls recycling of high affinity transporters to the cell membrane.

In contrast to the majority of the 23 amino acid permeases in yeast, which are constitutively expressed and import specific amino-acids with low affinity, high affinity permeases such as the general amino acid permease Gap1 and proline permease Put4 are highly expressed only during nitrogen limitation [16], [45], [46]. Gap1 and its related class of permeases have a high capacity for amino acid transport and are thought to scavenge amino acids for use as a source of nitrogen. Intracellular sorting is one of the mechanisms by which the quality of available nitrogen controls the presence of high affinity permeases at the cell membrane: during growth with a good nitrogen source such as ammonium, glutamate and glutamine, Gap1 is sorted to the vacuole for degradation [16]. When cellular nitrogen and amino acids levels are low, Gap1 is sorted to the plasma membrane. A complex consisting of Rps5, Bul1 and Bul2 ubiquitylates Gap1 and specifies its sorting to the multivesicular endosome. From the endosome, Gap1 can be targeted either to the vacuole or trafficked to the plasma membrane depending of the amino acid availability [47]. The amino acid-regulated step in this process appears to be Gap1 retrieval from the endosome rather than Gap1 ubiquitylation. Nevertheless, ubiquitylation is a prerequisite for controlling Gap1 localization because in its absence, Gap1 never reaches the endosome and is constitutively targeted to the plasma membrane. Therefore, loss of Bul2 function, such as in cells with the BY allele of BUL2, results in non-discriminatory import of amino acids and greater intracellular amino acid and nitrogen availability. Our finding that the common laboratory strain S288c carries a loss-of-function mutation in BUL2, subsequently leading to indiscriminant amino acid uptake, is important for future studies that exploit yeast as a model for amino acid sufficiency and TOR signaling. Specifically, such studies should include strains with wild-type BUL2; for example, they could employ the allele substitution strains described here. The mutation in BUL2 adds to the list of genetic alterations in the standard laboratory strain that are not representative of other members of the species such as loss of function changes in AMN1 [48] and MKT1 [49].

Similar to the control of Gap1, mammalian growth factor receptors are also regulated by ubiquitin-mediated trafficking. While yeast cells detect cellular resources directly through their import via permeases, multicellular organisms rely on growth factors such as IGF-1, which also stimulates TOR activity through Akt-Tsc-Rheb signaling, to coordinate nutrient availability with growth [1]. Cell surface localization of the IGF-1 receptor (IGF-1R) has been shown to depend on ubiquitylation by Nedd4, a homolog of the catalytic Rsp5 subunit of the Rsp5/Bul1/Bul2 ubiquitin ligase [50]. It is intriguing that *Nedd4^−/+^* mice have reduced IGF-1 receptors on the cell surface and phenotypes consistent with reduced IGF-1 signaling, including decreased body size [51], raising the possibility that they may share increased longevity with other IGF-1-related dwarf mice.

Reduced amino acid import in cells with functional Bul2 inhibits TORC1 activity, consistent with our observation of increased activity of TOR-inhibited transcription factor GLN3 in cells containing the RM BUL2 allele compared with cells which have the BY allele of BUL2. (In favorable nitrogen conditions, high TORC1 activity sequesters Gln3 in the cytoplasm.) Reduced TOR activity has been previously shown to extend both chronological and replicative lifespan in yeast [24], [25], [52]. Because reduced TOR activity extends lifespan also in higher eukaryotes [53]–[55], there is great interest in understanding the downstream events that mediate this effect.

Several mechanisms by which nutrients and TOR inhibition promotes CLS in yeast have been proposed, including reduced accumulation of acetate and/or acidification of culture media [14], promotion of respiration and autophagy [56], [57], and increased activity of stationary phase and stress-responsive transcription factors [25]. CLS experiments are often carried out in synthetic media which is complicated by significant media acidification due to release of organic acids during fermentation (the initial media pH of 4.2 decreases to <3 after cells reach stationary phase). A combination of acidic pH and high concentration of acetate in the media has been linked to reduction of cell viability [14]. Because our chronological aging assays are performed in rich media (YPD), which has an initial pH of 6.0 that reduces only to 5.8 after cells reach stationary phase, or buffered synthetic media, acetate toxicity is an unlikely mechanism for CLS modulation in our study.

A study by Bonawitz *et al.* linked reduction in TOR activity to increased cellular respiratory capacity [56]. While translation is generally inhibited by reduced TOR activity, Bonawitz *et al.* found that translation of mitochondrial proteins was enhanced and led to increased respiration during growth in glucose. Respiration becomes increasingly important for maintaining energy supplies and viability as cells transition from fermentative growth to stationary phase. The importance of respiration during the stationary phase transition is supported by the findings of two recent genome-wide studies that identified respiratory deficient mutants among those with the shortest CLS [15], [57]. In the same studies, mutants defective in autophagy, another process stimulated by TOR inhibition, were also found to have short CLS. These observations suggest that autophagy and respiration constitute important mediators by which reduced TOR activity promotes CLS.

The inhibition of TOR that occurs in cells during the post-diauxic shift and preparation for stationary phase also elicits specific transcriptional responses that are essential for maintaining viability during quiescence [25]. One target of TOR is the Rim15 protein kinase that translates nutrient limitation signals from TOR, as well as Ras/PKA and Sch9, into upregulation of cellular responses necessary for survival in stationary phase [58]. Similarly to Gln3, Rim15 is phosphorylated by the nutrient-sensing kinases and retained in the cytoplasm, but upon nutrient deprivation, dephosphorylated Rim15 translocates to the nucleus to activate transcription factors Gis1 and Msn2/4, which upregulate genes necessary for post-diauxic shift [59] and stress response respectively [60], [61]. Deletion of either RIM15 or its target transcription factors shortens CLS and abolishes benefits conferred by caloric restriction or mutations in Tor/Ras/Sch9 that mimic calorie restriction [25]. Since Rim15 and Gln3 are both directly regulated by TOR through cytoplasmic sequestration, we predicted that Gln3, like Rim15, would be essential for proper stationary phase transition and survival. In support of this idea, we have found that deletion of GLN3 in the vineyard strain dramatically shortens CLS (Figure S4) and that alteration of Bul2 function did not affect CLS in *gln3Δ* mutants. However, consistent with previous reports [24], [25], we found that deletion of GLN3 in the laboratory strain increased CLS. The paradoxical increase in CLS in response to GLN3 deletion in the laboratory strain is in opposition to the CLS detriment conferred by the loss of function of other transcription factors such as Msn2/4 or Gis1 which are, similarly to Gln3, upregulated during starvation. Furthermore, the opposing effect of GLN3 deletion in the laboratory and vineyard strains makes it difficult to determine the precise role of GLN3 as a mediator of CLS alterations in the cascade of events initiated by the BUL2 polymorphism.

Serving as a central link between nutrient availability and growth, TORC1 regulates many cellular processes including ribosome biogenesis, protein translation, autophagy and respiration [1]. During the examination of how telomere maintenance is affected by amino acid import, we discovered that ribonucleotide reductase (RNR) complex assembly during S-phase is modulated by the TOR-responsive transcription factor Gln3, defining a novel downstream role for TOR in DNA replication. We found that increased Gln3 activity, conferred by the deletion of URE2, upregulates Wtm1, which, in turn, promotes nuclear retention of the small RNR4 subunit in the nucleus. Deletion of WTM1 restores cytoplasmic localization of the small subunits and partially rescues the telomere length defect of *ure2Δ* cells. TORC1 inhibition by rapamycin was previously associated with genotoxic stress sensitivity and inability to maintain high Rnr1 and Rnr3 levels in response to DNA damage [62]. Using telomere length as a phenotype, we have uncovered a role of TORC1-responsive transcription factor GLN3 in modulation of RNR assembly during S-phase in response to cellular amino acid availability. TOR-mediated control of DNA replication adds further to TORC1's role in coordinating nutrient availability, growth and cell division.

What is the relevance of our observation to mammalian and human aging? Both dietary restriction and inhibition of TOR activity have been linked to lifespan extension in mice [40], [55]. At the same time, epidemiological studies in humans have found an association between longevity and long telomeres [9], [10]. Because our study demonstrates that dietary restriction and consequent reduction in TOR activity lead to reduction of telomere length, it will be important to determine whether reduced signaling in response to dietary restriction through this highly conserved nutrient and growth related pathway also reduces telomere length in mammals.

## Materials and Methods

### Yeast strains and media

Experiments were carried out using standard YPD media (2% glucose, 1% yeast extract, 2% peptone) unless otherwise noted (ie. ADCB assays). The strains used in this study, listed in Table S1, are from either the S288c (BY) or RM11-1A (RM) *S. cerevisiae* backgrounds. The segregant library has been previously described [11], except that AMN1 has been deleted in each of the segregants to facilitate single cell viability analysis. (The RM allele of AMN1 confers clumpiness, which precludes single cell analysis, whereas the S288c allele of AMN1 was previously shown to create a loss of AMN1 function [48]). Gene deletion mutants were either from yeast ORF deletion collection or were created using standard PCR transformation methods.

For allele replacement, we PCR-cloned a fragment containing 1 kb of the 3′ end of BUL2 and 1 kb BUL2 downstream sequence from either the BY or RM strain using a 5′ primer with an XhoI site (5′- GGCTCGAGGATTGATGATACCGCCAGCCAATCACC) and a 3′ primer with a HindIII site (3′- GGCCAAGCTTGCGGGAAAAAGGCCAAACTCTACG). These fragments were inserted between the XhoI and HindIII sites in pRS406, a vector containing URA3. We used site-directed mutagenesis (QuikChange II kit, Stratagene) to introduce the L883F polymorphism into the BY BUL2 vector. Allele replacement strains were generated using the “pop-in/pop-out” gene replacement method with the linearized BUL2 vector [63]. BUL2 allele replacement strains were first screened by sensitivity to ADCB and then PCR-sequenced to confirm the desired BUL2 polymorphisms.

### Microcolony assay for chronological aging

For each strain, 1 µL of saturated culture was inoculated into 150 µL of YPD (2% glucose) or buffered synthetic complete media [14] in 96-well plates. Plates were then incubated for 2 days at 30°C, at which point they were foil-sealed to prevent evaporation and kept at 30°C for the remaining time. Strains were examined in triplicate. To assay viability, 1 µl of each resuspended culture was harvested, diluted in water, spotted onto solid YPD media, and incubated for 24 hours at 30°C. Microcolonies and cells that had not divided were counted using a microscope, with the total number of events (n>200 for each culture) used as the denominator to determine viability percentage. Additionally, colony formation unit (CFU) assays was used to determine viability in select RM and BY strains. Comparison between CFU and microcolony values obtained show that the two assays are highly correlative (R = 0.98) (Figure S1).

### QTL mapping/genome-wide linkage analysis

Genome-wide linkage analysis of segregant data was performed using the publicly available R/qtl software. Effects of RM/BY allele inheritance in the segregants were examined using R (box plots) and Excel (student's t-test).

### ADCB toxicity assays

Initial ADCB toxicity assays were carried out using 25 µg/mL ADCB (L-Azetidine-2-Carboxylic Acid, Sigma-Aldrich) dissolved in SD media (1.9 g YNB, 0.5% (NH_4_)_2_SO_4_, 2% dextrose) supplemented with leucine (80 µg/mL), lysine (60 µg/mL), and uracil (20 µg/mL) to compensate for the auxotrophies present in the segregant library. Cells were inoculated into 150 µL media in 96-well plate and incubated at 30°C. Segregant growth in ADCB was quantified using absorbance at OD660 after 17 hours in 30°C. BUL2 allele replacement spot assays were carried out on solid SD media of the same composition with 25 µg/mL ADCB.

### Telomere length analysis

Genomic DNA was harvested from saturated 3 mL cultures using a phenol∶chloroform DNA extraction. Telomere lengths were evaluated as described in Gatbonton *et al.* [12]: genomic DNA was digested overnight with XhoI, resolved by gel electrophoresis (0.5% TBE, 0.9% agarose gel, run for 360 V•hr) and transferred to Hybond-N membrane. Terminal restriction fragments containing telomeres were visualized using ^32^P-labeled probes amplified from the Y′ subtelomeric sequence.

### Microarrays

Total RNA was harvested from 20 mL logarithmic phase cultures in biological triplicate using the hot phenol method previously described by Schmitt *et al.* [64]. Three competitive hybridizations for each experimental group (*ure2Δ* versus wildtype) were performed using three separate cultures, and the log_2_ of the expression ratio was calculated for every ORF. To assess the intrinsic variation of expression levels for different ORFs, wildtype versus pooled wildtype hybridizations were performed using three separate cultures. Arrays used were spotted oligo probe arrays generated by the Fred Hutchinson Cancer Research Center Genomics Resource. Probability of overlap with BUL2RM-upregulated transcripts was calculated using the binomial probability formula.

### Western blot analysis

Yeast whole cell extracts from 5 mL logarithmic phase cultures were harvested using the NaOH protein extraction method previously used by Thaminy *et al.* [65] and Kushnirov [66]. Proteins were resolved using SDS-PAGE (10% polyacrylamide gel, 120 V for 90 minutes) and transferred to a nitrocellulose membrane. Proteins of interest were probed with antibodies against actin (1∶1000 dilution, Neomarkers) or HA (1∶5000 dilution, Covance) and visualized using HRP-conjugated IgG antibodies (1∶1000, Vector Laboratories). Wtm1 blot intensity was quantified using ImageJ and normalized to actin intensity.

### Fluorescence microscopy

The Rnr4-GFP strain was obtained from the commercially available Invitrogen/UCSF GFP-tagged collection and genes were deleted using standard PCR transformation protocols. Cells from logarithmic phase cultures were harvested and fixed using paraformaldehyde, as previously described by Biggins *et al.* [67]. To visualize nuclei, fixed cells were incubated with 1 µg/mL DAPI for 1 hour, washed once and resuspended in sorbitol. Cells were sonicated before visualization and scoring. At least 200 events for both S-phase and G1 cells were scored for wildtype, *ure2Δ*, *wtm1Δ* and *ure2Δwtm1Δ* strains. At least 500 S-phase cells were scored for RM and BY BUL2 allele strains. Images were captured using a Nikon E800 fluorescence microscope.

## Supporting Information

Figure S1 Comparison of viability values obtained via the CFU assay versus the microcolony assay.
(TIF)

Figure S2 Comparison of phenotypes from telomere length and chronological aging genome-wide deletion screens. Telomere lengths of BY deletion strains from Gatbonton *et al.* [12] were plotted against their corresponding CLS from Fabrizio *et al.* [15]. Telomere length is indicated on the x-axis as −1,−2,−3 for mutants with shorter telomeres (shortened by by ≤50 bp, 50–200 bp, and ≥200 bp respectively) and as +1,+2,+3 for mutants with longer telomeres (longer by ≤50 bp, 50–200 bp, and ≥200 bp respectively). In this study, 72 mutants were identified as having short telomeres and 80 mutants with long telomeres. On the y-axis, we have plotted the fitness at day 11 of each deletion strain relative to the rest of the pool of ∼5000 strains from the deletion collection. (Strains were grown as pools and viability of each deletion strain is assessed at different timepoints as a ratio to the rest of the pool. Relative abundance of each strain at day 11 compared with their relative abundance at day 3 (t = 0) is taken as a measure of their relative fitness at day 11. For instance, a strain with a score of 1 has doubled its ratio of viable cells when compared to its ratio to the rest of the pool at day 3.) Of the roughly 600 strains identified as having putative altered longevity, either increased or decreased CLS, only a few also exhibit a telomere length defect. Conversely, most of the telomere length mutants have unremarkable CLS (most of the strains fall between −1 and 1 on the y-axis). Even the strains exhibiting altered telomere length and altered CLS did not fall into a set pattern: strains with telomere length defects, for both longer or shorter telomeres, were equally likely exhibit have increased or decreased viability.
(TIF)

Figure S3 Short telomeres conferred by deletion of URE2 are not epistatic with TLC1, YKU70 or TEL1. Southern blots show telomere length of single *ure2Δ* and double *ure2Δ tlc1Δ/yku70Δ/tel1Δ* mutants 25 doublings after germination of URE2/*ure2Δ* heterozygous diploids which are also TLC1/*tlc1Δ*, YKU70/y*ku70Δ*, or TEL1/*tel1Δ*. Telomere lengths of the double *ure2Δtlc1Δ*, *ure2Δyku70*, and *ure2Δtel1Δ* mutants are shorter than the telomere lengths of single *ure2Δ*, *tlc1Δ*, *yku70Δ*, or *tel1Δ* mutants.
(TIF)

Figure S4 CLS curves for *gln3Δ* and telomere length mutants. (A) Deletion of GLN3 extends lifespan in the BY parental strain, yet GLN3 deletion results in decreased lifespan in the RM parental background. Changes to Bul2 function, from either BUL2 allele replacement or BUL2 deletion, have no effect on lifespan in *gln3Δ* mutants in either parental background. (B) CLS analysis of *gln3Δ*, *ure2Δ*, *wtm1Δ* and *ure2Δgln3Δ* mutants in the BY background. (C) CLS analysis of mutants with long telomeres and short telomeres. We found no correlation between telomere length and chronological longevity.
(TIF)

Table S1 List of *S. cerevisiae* strains used in this study.
(XLS)

Table S2 Genomic loci linked to chronological lifespan.
(XLS)

Table S3 BUL2RM-upregulated transcripts overlap with those upregulated by amino acid deprivation, rapamycin treatment and loss of URE2. Transcripts upregulated in the segregants with the RM BUL2 allele and their corresponding expression level in cells undergoing amino acid deprivation, rapamycin treatment, or in *ure2Δ* mutant cells.
(XLS)

Table S4 *ure2Δ* transcript dataset. Transcripts which were upregulated 1.5-fold or more in *ure2Δ* cells relative to wildtype cells. The abundance of each transcript is presented as a log_2_ ratio relative to wildtype expression.
(XLS)
