# Supplementary figures and images for: Natural Polymorphism in BUL2 Links Cellular Amino Acid Availability with Chronological Aging and Telomere Maintenance in Yeast
Source: PLoS Genet. 2011 Aug 25;7(8):e1002250. doi: 10.1371/journal.pgen.1002250 (PMC3161923; doi:10.1371/journal.pgen.1002250)

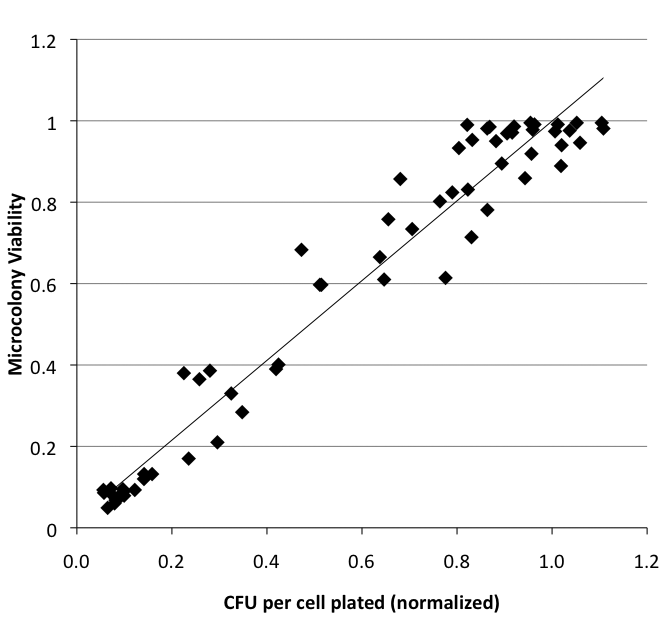

Supplement: Figure S1 — Comparison of viability values obtained via the CFU assay versus the microcolony assay. (TIF) [file pgen.1002250.s001.tif]

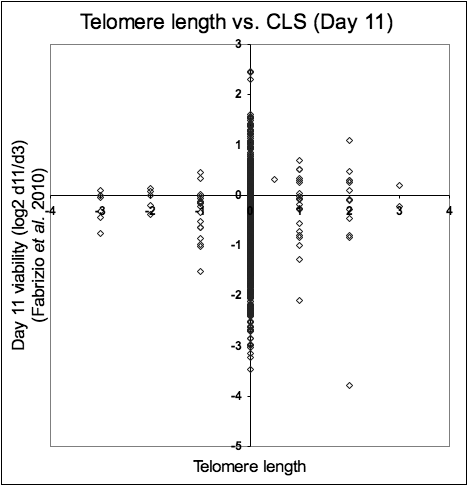

Supplement: Figure S2 — Comparison of phenotypes from telomere length and chronological aging genome-wide deletion screens. Telomere lengths of BY deletion strains from Gatbonton et al. [12] were plotted against their corresponding CLS from Fabrizio et al. [15]. Telomere length is indicated on the x-axis as −1,−2,−3 for mutants with shorter telomeres (shortened by by ≤50 bp, 50–200 bp, and ≥200 bp respectively) and as +1,+2,+3 for mutants with longer telomeres (longer by ≤50 bp, 50–200 bp, and ≥200 bp respectively). In this study, 72 mutants were identified as having short telomeres and 80 mutants with long telomeres. On the y-axis, we have plotted the fitness at day 11 of each deletion strain relative to the rest of the pool of ∼5000 strains from the deletion collection. (Strains were grown as pools and viability of each deletion strain is assessed at different timepoints as a ratio to the rest of the pool. Relative abundance of each strain at day 11 compared with their relative abundance at day 3 (t = 0) is taken as a measure of their relative fitness at day 11. For instance, a strain with a score of 1 has doubled its ratio of viable cells when compared to its ratio to the rest of the pool at day 3.) Of the roughly 600 strains identified as having putative altered longevity, either increased or decreased CLS, only a few also exhibit a telomere length defect. Conversely, most of the telomere length mutants have unremarkable CLS (most of the strains fall between −1 and 1 on the y-axis). Even the strains exhibiting altered telomere length and altered CLS did not fall into a set pattern: strains with telomere length defects, for both longer or shorter telomeres, were equally likely exhibit have increased or decreased viability. (TIF) [file pgen.1002250.s002.tif]

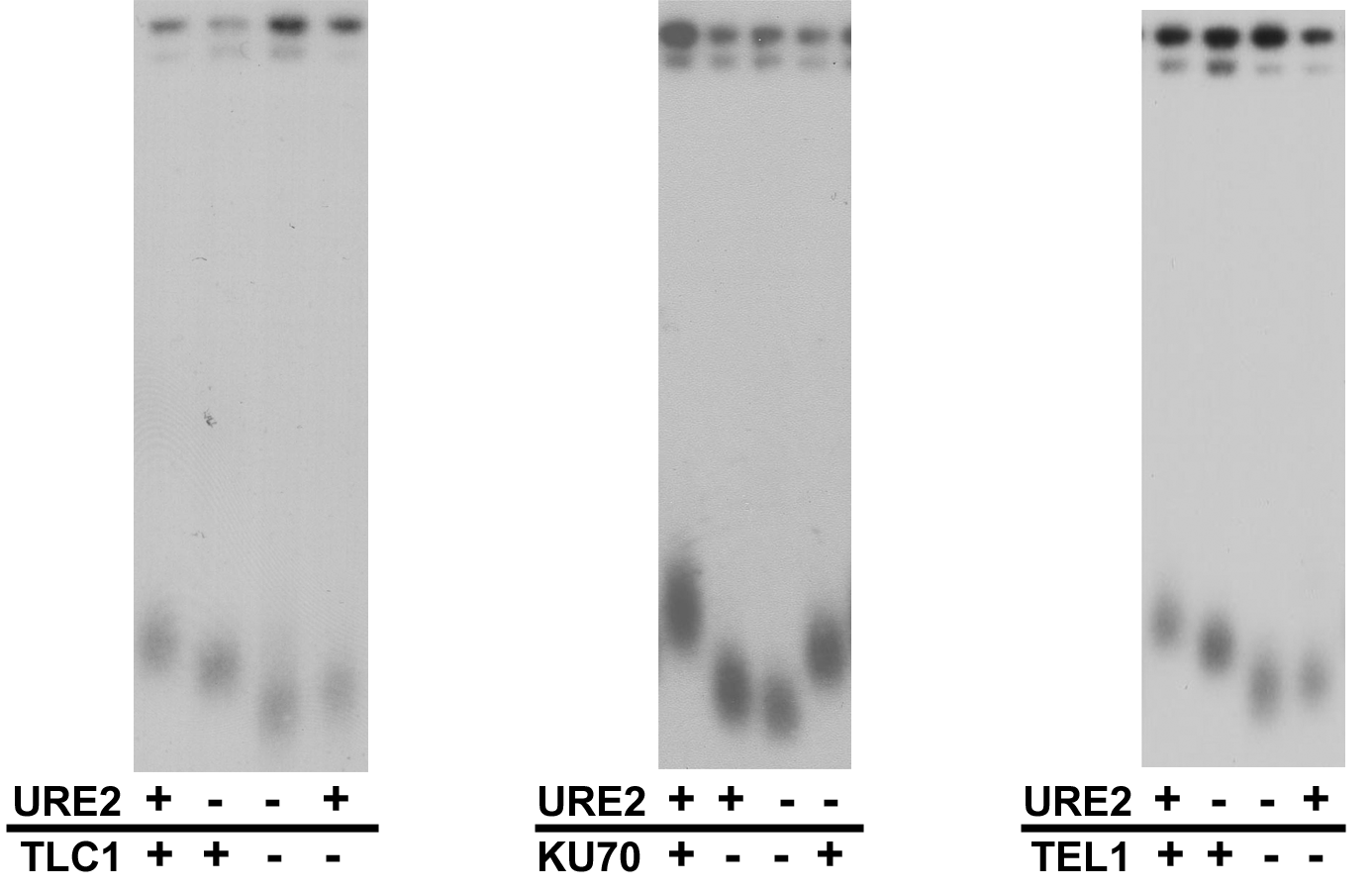

Supplement: Figure S3 — Short telomeres conferred by deletion of URE2 are not epistatic with TLC1, YKU70 or TEL1. Southern blots show telomere length of single ure2Δ and double ure2Δ tlc1Δ/yku70Δ/tel1Δ mutants 25 doublings after germination of URE2/ure2Δ heterozygous diploids which are also TLC1/tlc1Δ, YKU70/yku70Δ, or TEL1/tel1Δ. Telomere lengths of the double ure2Δtlc1Δ, ure2Δyku70, and ure2Δtel1Δ mutants are shorter than the telomere lengths of single ure2Δ, tlc1Δ, yku70Δ, or tel1Δ mutants. (TIF) [file pgen.1002250.s003.tif]

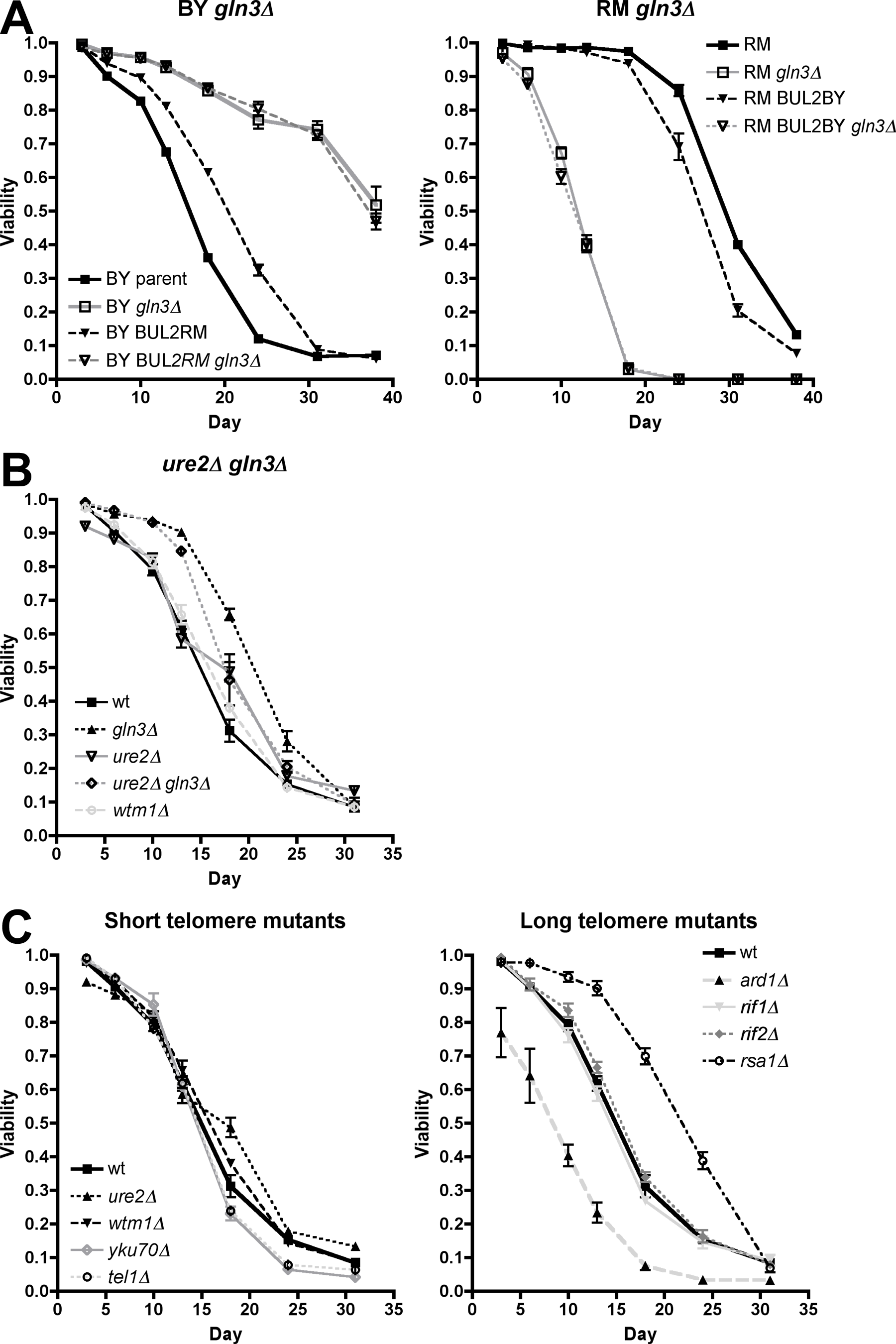

Supplement: Figure S4 — CLS curves for gln3Δ and telomere length mutants. (A) Deletion of GLN3 extends lifespan in the BY parental strain, yet GLN3 deletion results in decreased lifespan in the RM parental background. Changes to Bul2 function, from either BUL2 allele replacement or BUL2 deletion, have no effect on lifespan in gln3Δ mutants in either parental background. (B) CLS analysis of gln3Δ, ure2Δ, wtm1Δ and ure2Δgln3Δ mutants in the BY background. (C) CLS analysis of mutants with long telomeres and short telomeres. We found no correlation between telomere length and chronological longevity. (TIF) [file pgen.1002250.s004.tif]
